# The Multiplication of Metropolitan Hospitals

**Published:** 1892-08-13

**Authors:** Richard Kershaw

**Affiliations:** Secretary of the Central London Throat and Ear Hospital, Gray's Inn Road


### HOSPITAL OPINION.

[Contributions critical, suggestive, controversial, and technical are invited for this column, which is open to everyone who has anything to say that is of practical value and interest.]

### THE MULTIPLICATION OF METROPOLITAN HOSPITALS.

Mr. Richard Kershaw, Secretary of the Central London Throat and Ear Hospital, Gray's Inn Road, writes: Permit me to offer a few remarks upon the article from your correspondent under this heading in your issue of July 23rd.

Naturally, I am chiefly concerned in that portion wherein he alludes to the foundation of this hospital. I will at once endeavour to establish the *raison d'être* of its existence, and at the same time shall hope to prove how fallacious are the comments made by your correspondent.

The Central London Throat and Ear Hospital was founded in 1874, with a properly constituted committee of management, and issuing an annual public report, with a professionally audited half-yearly balance-sheet. At that time the only other hospital existing for the treatment of diseases of the throat, and similarly constituted, was the Throat Hospital in Golden Square, established only nine years earlier, not twenty years. I may also remark that this institution is the only one amongst those considered unnecessary by your correspondent that receives a grant from the Hospital Sunday Fund, and this grant has been awarded from the very first.

But very few of the general hospitals, at the time of our foundation in 1874, had special departments for diseases of the ear, and still fewer for the throat, but even if such departments were in existence, the patients there treated would be numbered by tens, whereas those who have sought aid at our institution have from the first been counted by thousands. In view of these facts it cannot be contended that there was no need for such an institution as this one.

I may refer your correspondent to the work entitled "Pay Hospitals and Paying Wards throughout the World," by Mr. Henry C. Burdett, published in 1879. The author, as the result of a personal visit to this hospital—then established five years—describes the work done, but he did not consider that the institution was unnecessary. To come to a more recent date, Mr. Burdett, in giving his evidence before the Lords' Committee on Hospitals, said, in answer to Question 28541 (pertaining to the provident system at special hospitals), that the Central London Throat and Ear Hospital appears to be doing good among that class of patients who alone ought to receive advice for a nominal sum. Even these facts may not satisfy your correspondent as to the "need" for this institution. I may, therefore, draw his attention to my letter which you published in your issue of July 9th, in which I remarked that during the last ten years no fewer than 1,934 medical practitioners have sought and received instruction in those diseases which it is the province of this hospatal to treat, while in the past year 1,000 patients were sent by medical men from all parts of London and the provinces. It must be obvious that the reason why so many young qualified medical men attend the practice of this special hospital is that they believe they receive a more thorough system of instruction than is to be found at the general hospital in which they have received that amount of medical education which has qualified them for their diplomas.

It seems to me to be very unfair criticism to doubt the necessity for this hospital, established close upon twenty years, without also examining the *raison d'être* of all others. I presume that the Hospital for Paralysis in Regent's Park, founded only seven years after the National Hospital for Paralysis in Queen Square, was established to meet a want, and that the Chelsea Hospital for Women, founded in 1871, was called for in spite of the fact that in that year there were already in existence five similar institutions; also that there was need for the Royal Westminster Ophthalmic Hospital, founded in 1816, although the Royal London Ophthalmic Hospital was established but a few years earlier. Will it even be advanced that there is no necessity for the five ophthalmic hospitals now existing? I find that no less than 3,967 in-patients and 53,847 out-patients were treated by these institutions last year alone. And what about the numerous chest hospitals? I have no doubt that the other institutions which your correspondent does not consider necessary will be well able to take care of themselves, but when one considers that Dr. Bristowe, F.R.S., President of the Hospitals Association, became the Consulting Physician to the London Skin Hospital, established only four years ago, within a hundred yards of another skin hospital, which had been in existence for thirty years, it must be evident that he considered there was a necessity for such an institution. This physician has quite recently also joined the staff of the West End Hospital for Diseases of the Nervous System.

The London Throat Hospital, founded in 1887, is to be found amongst those institutions the staff of which delivers post graduate lectures, which are presided over by Mr. Jonathan Hutchinson, F.R.S., ex-President of the Royal College of Surgeons.

